# A new species group in the genus *Dichaetophora*, with descriptions of six new species from the Oriental region (Diptera, Drosophilidae)

**DOI:** 10.3897/zookeys.665.11609

**Published:** 2017-04-04

**Authors:** Jin-Hua Yang, Masanori J. Toda, Awit Suwito, Rosli Hashim, Jian-Jun Gao

**Affiliations:** 1 State Key Laboratory for Conservation and Utilization of Bioresources in Yunnan, Yunnan University, 2 Cuihubeilu, Kunming 650091, China; 2 Hokkaido University Museum, Hokkaido University, N10, W8, Kita-ku, Sapporo 060-0810, Japan; 3 Zoology Division (Museum Zoologicum Bogoriense), Research Center for Biology-LIPI, Cibinong, Bogor 16911, Indonesia; 4 Institute of Biological Science, University of Malaya, 50603 Kuala Lumpur, Malaysia

**Keywords:** DNA barcoding, geographical isolation, mitochondrial DNA, taxonomy

## Abstract

The genus *Dichaetophora* Duda comprises 61 described species classified into four species groups: *agbo*, *tenuicauda*, *acutissima* and *sinensis*. This genus is distributed exclusively in the Old World, and is rich in species in the tropical and subtropical areas of the Oriental, Australasian, and Afrotropical regions. In this paper, a new species group, the *trilobita* group, is established for six new species discovered from the Oriental region. The delimitation of these species is firstly performed in light of morphology and further with the aid of DNA sequences of the mitochondrial *COI* and *COII* (cytochrome *c* oxydase, subunits I and II, respectively) genes, considering also their respective geographical origins. Then, the new species (*trilobita* Yang & Gao, **sp. n.**, *heterochroma* Yang & Gao, **sp. n.**, *flatosternata* Yang & Gao, **sp. n.**, *borneoensis* Yang & Gao, **sp. n.**, *javaensis* Yang & Gao, **sp. n.**, and *sumatraensis* Yang & Gao, **sp. n.**) are described, and a key, based on not only morphological but also molecular information, is provided.

## Introduction

The genus *Dichaetophora* is widely distributed in the Old World, especially its tropical and subtropical regions. This genus was originally established by Duda (1940) as a subgenus in the genus *Drosophila* Fallén, for the Seychellean species, *Drosophila aberrans* Lamb. Burla (1954) supplemented three African new species (including an informally named one) into *Dichaetophora* and revised its diagnostic characters. Since then, the species composition of this subgenus [genus since Grimaldi’s (1990) upgrading] had been altered time and again in relation to the genus *Nesiodrosophila* Wheeler & Takada (see Hu and Toda 2002). Hu and Toda (2002) examined the relationships among the genera *Dichaetophora*, *Nesiodrosophila*, the *Lordiphosa tenuicauda* species group and some presumably related genera by a cladistic analysis of morphological characters. As a result, the revised and enlarged genus *Dichaetophora* was proposed and subdivided into three species groups, i.e., the *agbo*, *acutissima* and *tenuicauda* groups. Hu and Toda (2005) established the forth (*sinensis*) species group for four species newly described from China, raising the number of known *Dichaetophora* species to 61. In the present study, a new species group is established for six new species of *Dichaetophora* recently discovered from the Oriental region, the *trilobita* group. The species delimitation is based on not only morphological but also geographical and DNA sequence data. A key to the six species is provided.

## Materials and methods

### Specimens

A summary of the specimens employed in the present study is shown in Table 1. The flies were mostly captured by net sweeping on herbs growing along watersides in open forests or at forest edges. Specimens were preserved in either 70% (after fixing with Kahle’s solution for morphological observation) or 100% ethanol (for DNA sequencing).

**Table 1.** Summary of new species and specimens of *Dichaetophora* employed in the present study.

Code of morpho-species	Formal name	Voucher # ^a^	Distribution / collection site	Collection date
sp.K1	*trilobita* sp. n.	#03876 (♂)	Park Headquarters, Mt. Kinabalu, Sabah, Malaysia	11.iii.2008
		#**03877**–8 (♂, ♀)	Ulu Gombak, Selangor, Malaysia	8.xii.2013
		#03882 (♀)	Poring, Mt. Kinabalu, Sabah, Malaysia	20.iii.2008
		unnumbered (1♂, 1♀)	Kubah, Sarawak, Malaysia	19.i.1999
sp.K2	*heterochroma* sp. n.	#**03879**–81 (2♂, 1♀), (1♂)	Poring, Mt. Kinabalu, Sabah, Malaysia	20.iii.2008
		#03883 (♀)	Poring, Mt. Kinabalu, Sabah, Malaysia	13.iii.2008
		#03884–6 (1♂, 2♀)	Ulu Senagang, Crocker Range, Sabah, Malaysia	18.x.1999
		unnumbered (1♂)	Poring, Mt. Kinabalu, Sabah, Malaysia	19.iii.2008
		unnumbered (1♂)	Poring, Mt. Kinabalu, Sabah, Malaysia	3.x.1999
		unnumbered (3♀)	Mahua, Crocker Range, Sabah, Malaysia	14.x.1999
sp.K2-like	*flatosternata* sp. n.	#04171–8 (6♂, 2♀)	Guanlei, Xishuangbanna Nature Reserve, Yunnan, China	14–15.x.2012
sp.K3	*borneoensis* sp. n.	#03893–4 (♀)	Park Headquarters, Mt. Kinabalu, Sabah, Malaysia	11.iii.2008
		#**03895** (♂)	Park Headquarters, Mt. Kinabalu, Sabah, Malaysia	16.viii.2011
		#03896 (♂)	Park Headquarters, Mt. Kinabalu, Sabah, Malaysia	17.viii.2011
		unnumbered (8♂, 4♀)	Park Headquarters, Mt. Kinabalu, Sabah, Malaysia	2.i.1999
		unnumbered (1♂)	Poring, Mt. Kinabalu, Sabah, Malaysia	28.xii.1998
		unnumbered (1♂)	Mahua, Crocker Range, Sabah, Malaysia	14.x.1999
	*javaensis* sp. n.	#03887–89 (♀)	Cikaniki, Mt. Halimun, West Java, Indonesia	6.xi.2009
		#**03892** (♂)	Cikaniki, Mt. Halimun, West Java, Indonesia	7.xi.2009
		unnumbered (1♀)	Cikaniki, Mt. Halimun, West Java, Indonesia	10.xi.2009
		unnumbered (1♂)	Cibodas, West Java, Indonesia	16.xi.2013
		unnumbered (1♂)	Mt. Patuha, Sugihmukti, West Java, Indonesia	13.x.2004
	*sumatraensis* sp. n.	#**03890**–1 (♂, ♀)	Mt. Kerinci, Jambi, Sumatra, Indonesia	7.x.2004
		unnumbered (1♀)	Mt. Kerinci, Jambi, Sumatra, Indonesia	6.x.2004

^a^ Numbers in bold indicate holotypes of new species.

### Species delimitation

**Figure 1.**
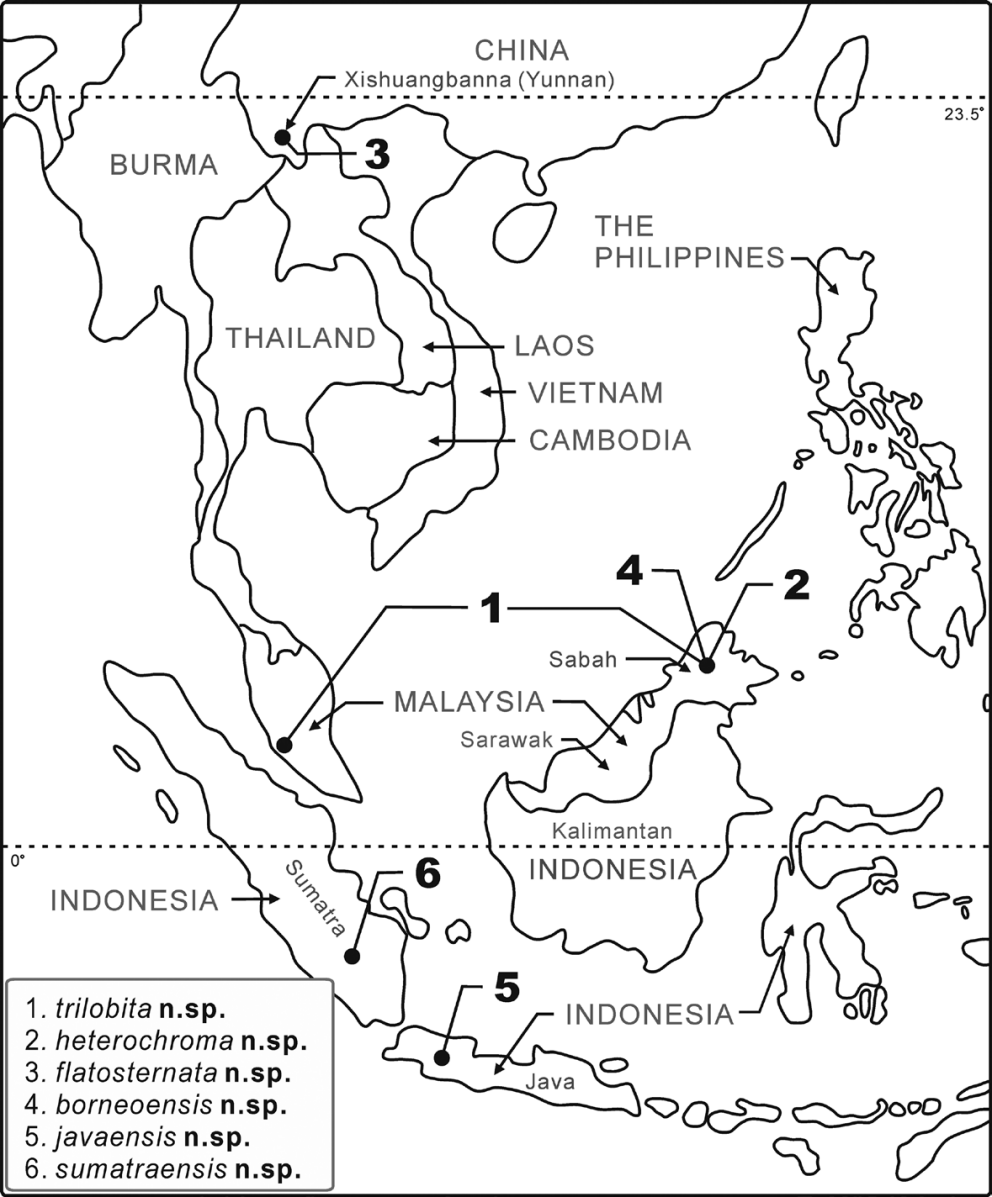
Geographical distribution of the *Dichaetophora trilobita* species group. See the text for the detailed information of the collection sites on the map.

The specimens were first identified as of *Dichaetophora* in light of morphology referring to Hu and Toda’s (2005) diagnosis of this genus. Then, they were examined for external morphology, morphometric characters and detailed structures of some dissected organs by the same methods as in Li et al. (2014), and sorted into morpho-species. For each of these morpho-species, representative specimens suitable for DNA sequencing were selected, considering also the numbers, geographical origins, and genders of available specimens. For each of the selected specimens, the total DNA was extracted from a hind-leg (usually the right one) or small piece(s) of abdominal tissue picked from the dissection cut of terminalia, using the TIANamp® Genomic DNA Kit. DNA sequences of the 658-bp barcoding region of the mitochondrial *COI* (cytochrome *c* oxydase subunit I) gene were then amplified and sequenced with the Folmer primers (Folmer et al. 1994; Table 2), using the same PCR cycle program as in Li et al. (2014). In addition, we determined the DNA sequences of the whole 688-bp region of the mitochondrial *COII* (cytochrome *c* oxidase subunit II) gene, using the primer pair designed by Simon et al. (1994; Table 2), with the same PCR cycle program used in Gao et al. (2007). The sequences obtained were edited in the SeqMan module of the DNAStar package, version 7.1.0 (DNAStar, Inc., Madison, WI), and aligned in MEGA7 (Kumar et al. 2016). We performed a tree-based DNA barcoding with the *COI* and *COII* sequences, respectively, with Bayesian trees constructed using MrBayes 3.1 (Ronquist and Huelsenbeck 2003). For this, the sequence alignment of each gene was partitioned into two subsets (codon positions 1 plus 2, and codon position 3), with choice of substitution models justified via model testing (Srivathsana and Meier 2012) in MEGA7 using the Bayesian Information Criterion. In Bayesian inference, sampling frequency was set as every 1000 generations, and numbers of chains = 4. Two analyses were run simultaneously till the average deviation of split frequencies fell well below 0.01. Therefore, in all analyses, full runs of 5,000,000 generations were performed. In each analysis, 1000 early-phase samples were discarded as burn-in for each run, yielding a total of 8,002 trees to construct a 50% majority consensus tree with nodes characterized by posterior probability (PP). We then summarized the information of intra- and interspecific p-distances calculated without data partitioning. The morpho-species were then reconsidered by integrating information from the morphology, the geographical distribution (Fig. 1) and DNA barcodes.

**Table 2.** Primer sequences for PCR/sequencing.

Target region	Primer name	Primer sequence (5’–3’)	Reference
*COI*	LCO1490	GGTCAACAAATCATAAAGATATTGG	Folmer et al. (1994)
	HCO2198	TAAACTTCAGGGTGACCAAAAAATCA	ditto
*COII*	COII-1	ATGGCAGATTAGTGCAATGG	Simon et al. (1994)
	COII-2	GTTTAAGAGACCAGTACTTG	Ditto

### Descriptions

In species illustration, a DinoLite® Digital Eyepiece Camera was used to microphotograph some organs for representative specimens. McAlpine (1981) was followed for the morphological terminology, and Zhang and Toda (1992) for the definitions of measurements and indices. The examined specimens are deposited in the following institutes:

**UMKL** Zoological Museum, Institute of Biological Science, University of Malaya, Kuala Lumpur, Malaysia

**KPSP** Kinabalu Park, Sabah Parks, Sabah, Malaysia

**ZSM** Institute for Tropical Biology and Conservation, Universiti Malaysia Sabah, Kota Kinabalu, Sabah, Malaysia

**MZB** Museum Zoologicum Bogoriense, Bogor, Indonesia

**SEHU** Systematic Entomology, The Hokkaido University Museum, Hokkaido University, Sapporo, Japan

**KIZ** Kunming Natural History Museum of Zoology, Kunming Institute of Zoology, Chinese Academy of Sciences, Kunming, China

## Results

### Species delimitation

**Figure 2.**
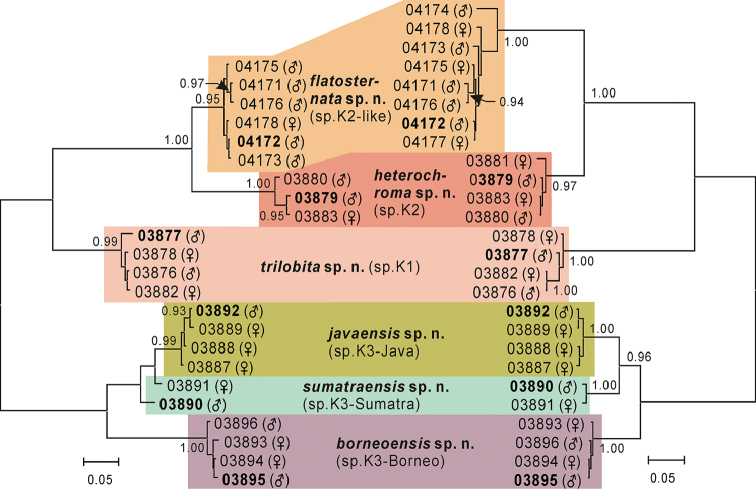
Bayeisan trees deduced with *COI* (left) and *COII* (right) gene sequences. Label of each operational taxonomic unit (OUT) is given in the format of “voucher number (sex)”. Numbers beside nodes are posterior probabilities (when ≥ 0.90). Bold voucher numbers indicate holotype specimens.

The specimens examined were first sorted into four morpho-species (Table 1). We got 23 *COI* and 26 *COII* sequences. The GenBank accession numbers are KY809802−KY809824 for the *COI*, and KY809825−KY809850 for the *COII* sequences. Table 3 shows the result of model selection. The *COI* and *COII* Bayesian trees (both unrooted) are shown in Fig. 2. Each of the morpho-species sp.K1 [from Peninsular Malaysia and Borneo (Sarawak and Sabah)], sp.K2 (Sabah) and sp.K2-like (Xishuangbanna, Yunnan, southwestern China) was strongly suggested to be monophyletic. Sp.K2 and sp.K2-like formed a well-supported clade (PPs = 1.00 in both of the *COI* and *COII* trees, respectively). Sp.K1, which is sympatric with sp.K2 in Sabah, formed a clade independent from the clade of sp.K2+sp.K2-like. Thus, these three morpho-species, spp.K1, K2 and K2-like, were recognized as independent species, i.e., *trilobita* sp. n., *heterochroma* sp. n. and *flatosternata* sp. n., respectively.

Specimens of the morpho-species sp.K3 clustered into three more or less diverged, allopatric lineages each endemic to Borneo (Sabah), West Java, or Sumatra (Jambi) in the *COII* tree (PP = 1.00 for each lineage). While the former two lineages were recovered in the *COI* tree (PPs = 0.99 and 1.00, respectively), the last one was not supported in this tree. Table 4 shows the summary of intra- and interspecific p-distances for the six putative species. The interspecific mean p-distances for *COI* sequences among these three lineages of sp.K3 varied from 0.0349 (Java vs. Sumatra) to 0.0751 (Borneo vs. Java), coinciding with the smallest interspecifc distance variability of 5.9±4.1% (uncorrected divergence) for *COI* sequences in Diptera (Meier et al. 2008) and being larger than their intraspecific mean distances ranging from 0.0045 (Java) to 0.0185 (Sumatra). However, these lineages are morphologically very similar, differing from each other in so few morphological characters that it is hard to distinguish between them (see descriptions). On the other hand, comparison of the *COI* and *COII* nucleotide sequences among these lineages has revealed that there are fixed, lineage-specific nucleotides at more than one sites, where nucleotides remain unchanged in the other three species (Table 5). Such sites can therefore be used as pure molecular diagnostic characters (Sarkar et al. 2002, DeSalle et al., 2005) for respective lineages. Taking into account their geographically isolated situations as well, we regard these lineages as three independent, cryptic species, i.e., *borneoensis* sp. n., *javaensis* sp. n. and *sumatraensis* sp. n.

**Table 3.** Models selected for sequence subsets ^a^.

Gene	Data set	Model selected	BIC score	ln *L*	Invariant	Gamma	*R*
*COI*	whole	GTR+G	4417.9362	-1962.0299	n/a	0.2057	2.1514
	CP_1+2_	K2+G	2057.0568	-823.9557	n/a	0.0500	18.7827
	CP_3_	T92+G	2322.3495	-967.9958	n/a	1.0658	3.3825
*COII*	whole	T92+G	4745.1717	-2118.2231	n/a	0.1231	4.6468
	CP_1+2_	T92+G	2250.9406	-881.6321	n/a	0.0500	4.4687
	CP_3_	T92+G	2525.5613	-1037.0171	n/a	0.9274	9.6084

^a^ Abbreviations: CP_1+2_, codon positions 1 plus 2; CP_3_, condon position 3; GTR, general time reversible; G, discrete Gamma distribution; K2, Kimura 2-parameter; T92, Tamura 3-parameter; BIC score, Bayesian Information Criterion score; ln*L*, maximum likelihood value; Invariant, estimated fraction of invariant sites; Gamma, gamma shape parameter; *R*, estimated value of transition/transversion bias.

**Table 4.** Summary of intra- and interspecific p-distances.

Species (Morpho-species code)	Intraspecific mean distance (±SE) ^a^		Interspecific mean distance (*COI* / *COII*) ^b^					
	*COI*	*COII*	1	2	3	4	5	6
1. *trilobita* sp. n. (sp.K1)	0.0078 ± 0.0028	0.0098 ± 0.0033		0.0112 / 0.0117	0.0101 / 0.0098	0.0127 / 0.0128	0.0122 / 0.0115	0.0123 / 0.0114
2. *heterochroma* sp. n. (sp. 2)	0.0076 ± 0.0026	0.0044 ± 0.0018	0.1123 / 0.1054		0.0082 / 0.0076	0.0128 / 0.0103	0.0114 / 0.0104	0.0125 / 0.0114
3. *flatosternata* sp. n. sp.K2-like)	0.0041 ± 0.0017	0.0111 ± 0.0021	0.1105 / 0.1148	0.0573 / 0.0707		0.0128 / 0.0112	0.0127 / 0.0110	0.0123 / 0.0106
4. *borneoensis* sp. n. (sp.K3, Borneo)	0.0063 ± 0.0024	0.0007 ± 0.0007	0.1256 / 0.1273	0.1412 / 0.1159	0.1448 / 0.1355		0.0082 / 0.0075	0.0085 / 0.0076
5. *javaensis* sp. n. (sp.K3, Java)	0.0045 ± 0.0019	0.0044 ± 0.0020	0.1118 / 0.1170	0.1295 / 0.1136	0.1271 / 0.1280	0.0751 / 0.0607		0.0079 / 0.0072
6. *sumatraensis* sp. n. (sp.K3, Sumatra)	0.0185 ± 0.0065	0.0060 ± 0.0027	0.1007 / 0.1178	0.1184 / 0.1129	0.1175 / 0.1223	0.0713 / 0.0576	0.0349 / 0.0468	

^a^ SE, standard error; ^b^ Values of p-distance below diagonal, values of standard error above diagonal.

### Taxonomy

In the following descriptions of the new species group and new species, and also the key to species, some figures in Hu and Toda (2005) are referred to, with their original numbers given in double quotation marks.

The six new species to be described here certainly belong to the genus *Dichaetophora*, according to its diagnosis revised by Hu and Toda (2002): cibarium only slightly protruded at anterolateral corners; oviscapt with apical ovisensillum robust and largest, distinguishable from the others; basal lobe of palpus without setulae; hypopharyngeal apodeme expanded anteriorly; labellum with less than six pseudotracheae; ocellar setae outside triangle made by ocelli. Within *Dichaetophora*, they should be related to the *sinensis* group, sharing some characters regarded by Hu and Toda (2005) as diagnostic for the latter group: very large ocellar triangle (“Fig. 1”); large number (≥ 40 per side) of medial sensilla on cibarium (“Fig. 6”); ventral surface of prementum forming discrete bump (Fig. 4E, J, O, T, Y, D’; “Figs 8–11A”). However, they lack some other diagnostic characters of the *sinensis* group: foreleg tibia with stout apical seta distinctly thicker than preapical dorsal seta (“Fig. 2”); aedeagus apically with membranous, trumpet-like dilation (“Figs 8F, 9–11D”). And, three of them, *trilobita* sp. n., *heterochroma* sp. n. and *flatosternata* sp. n., share a particular character, i.e., 4 pseudotracheae varying in thickness (“Fig. 4”), with the *agbo* species group (Hu and Toda 2005). Furthermore, all the six new species possess some characters specific to themselves: there are two or three prominent setae on the anteromedial portion of cercus (Figs 5–10B); the cercus is strongly sclerotized along the anterior to caudoventral margin, which seems to be homologous with the strong sclerotization of the caudoventral portion of cercus seen in three species of the *sinensis* group (“Figs 9–11B”), but the sclerotized plates of cerci are fused with each other caudoventrally and to epandrium anteroventrally (Figs 5–10A,B). Based on these morphological characteristics, we establish a new species group, the *trilobita* species group, in *Dichaetophora*, for the six new species.

### *Dichaetophora trilobita* species group

**Diagnosis.** Cercus anteromedially with two or three prominent setae on anteromedial portion, strongly sclerotized along anterior to caudoventral margin; sclerotized plates of curci fused with each other caudoventrally and to epandrium anteroventrally (Figs 5–10A, B).

**Common characters.** *Head* (Fig. 4): Eye red, with dense interfacetal setulae; longest axis nearly orthogonal to body axis. Frons, face, gena, occiput, postgena and clypeus glossy black; facial carina blackish brown. Ocellar very large, nearly rectangular, anteriorly reaching to ptilinal fissure; frontal vitta narrow, without interfrontal setulae. Pedicel grayish brown; arista with 6−7 dorsal and 2−3 ventral branches in addition to terminal fork. Subvibrissal seta not differentiated, as small as other orals. Palpus slender, apically with one prominent ventral and one subprominent dorsal setae. Cibarium not thickened on anterior margin, with four anterior sensilla arranged square; dorsal wall pear-shaped, anteriorly somewhat dilated in dorsal view and strongly convex in lateral view; anterior portion of hypopharynx shorter than posterior tubular portion. Prementum with 5–6 (one proximal, one central, 2–3 lateral, and one distal longest) pairs of setae. Labellum with four pseudotracheae per side.

**Figure 3.**
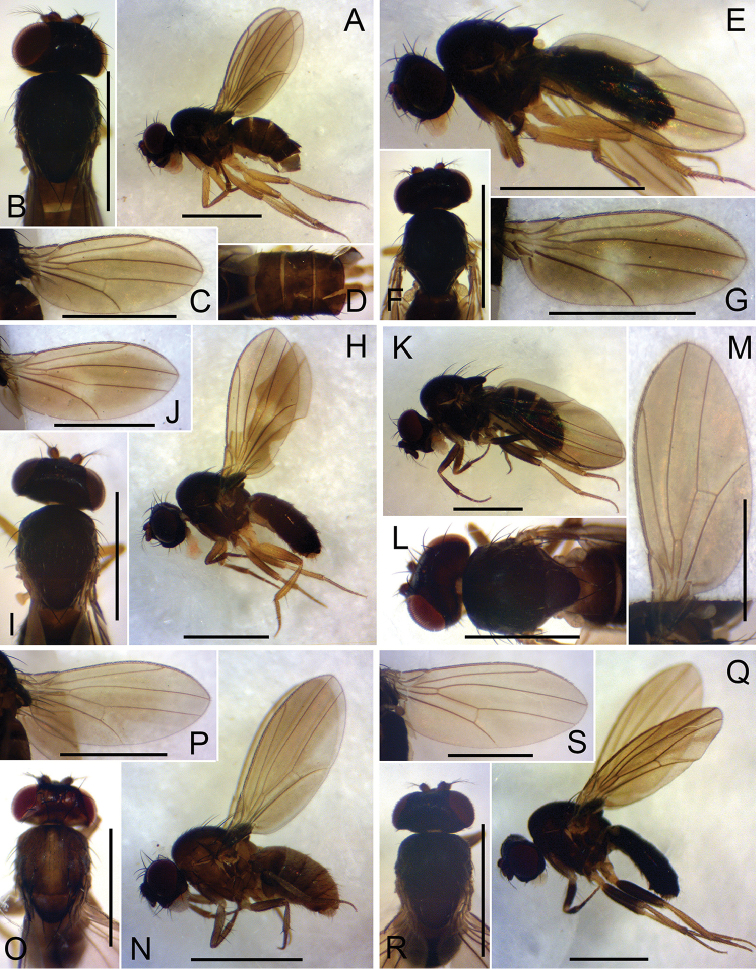
Left lateral habitus, head and thorax (dorsal view), wing (left, ventral view), and abdomen (dorsal view). **A−D** *Dichaetophora tirlobita* sp. n. (#03877) **E−G** *D. heterochroma* sp. n. (#03879) **H−J** D. flatosternata sp. n. (#04172) **K−M** *D. borneoensis* sp. n. (#03895) **N−P** *D. javaensis* sp. n. (#03892) **Q−S** *D. sumatraensis* sp. n. (#03890). Scale bars: 1.0 mm.

*Thorax* (Fig. 3A, B, E, F, H, I, K, L, N, O, Q, R): Scutum and scutellum matt, entirely black. Postpronotum, thoracic pleura and notopleural portion blackish brown to black. Acrostichal setulae in six rows. Mid katepisternal seta minute, indistinguishable from a few underneath others.

*Legs* (Fig. 3A, E, H, K, N, Q): Preapical dorsal setae present on all tibiae; foreleg apical seta as thick as preapical dorsal one. Foreleg first tarsomere slightly shorter than total length of four succeeding tarsomeres; all tarsi narrowing distally, with small, apical claws.

*Abdomen* (Fig. 3A, D, E, H, K, N, Q): Tergites blackish brown. Sternites grayish yellow.

*Male terminalia* (Figs 5A–H, 6A–I, 7A–I, 8A–G, 9A–G, 10A–H): Surstylus basally narrowly fused to epandrium, with 8–10 peg-like prensisetae on caudal margin. Hypandrium pale brown, submedially with a pair of small, pubescent lobes apically bearing short paramedian seta. Paramere broad, plate-like, not pubescent, partly fused to hypandrium, articulated with aedeagus, with two sensilla. Aedeagus apically without pale, membranous trumpet-like dilation; basal processes absent; aedeagal guide present, apically fused to hypandrium; apodeme as long as aedeagus.

*Female terminalia* (Figs 5I–K, 6J–L, 7J–L, 8H–J, 9H–J, 10I–K): Oviscapt valve yellowish brown, apically pointed and slightly bent outward, with four (three dorsal, one ventral) subterminal, trichoid ovisensilla.

**Included species.** *trilobita* Yang & Gao, sp. n., *heterochroma* Yang & Gao, sp. n., *flatosternata* Yang & Gao, sp. n., *borneoensis* Yang & Gao, sp. n., *javaensis* Yang & Gao, sp. n., and *sumatraensis* Yang & Gao, sp. n.

**Table 5.** Selected diagnostic nucleotide sites for each of *borneoensis* sp. n., *javaensis* sp. n., and *sumatraensis* sp. n. in the *COI* and *COII* sequences. For nonsynonymous nucleotide substitutions, corresponding amino acid status are given in parentheses. Hyphens (-) indicate missing data, and dots (.) indicate identical symbols with the first sequence (i.e., that of the specimen #03876 of *trilobita* sp. n.).

Species	Voucher #	Diagnostic sites																		
		*COI*								*COII*										
		142	205	235	499	519	532	541	544	18	69	270	303	309	381	389	393	441	513	636
*trilobita* sp. n.	#03876	T	T	T	T	C (Ala)	A	T	T	T	T	T	T	A	A	C (Thr)	T	T	G	A
	#03877	.	.	.	.	. (.)	.	.	.	.	.	.	.	.	.	. (.)	.	.	.	.
	#03878	.	.	.	.	. (.)	.	.	.	.	.	.	.	.	.	. (.)	.	.	.	.
	#03882	.	.	.	.	. (.)	.	.	.	.	.	.	.	.	.	. (.)	.	.	.	.
*heterochroma* sp. n.	#03879	.	.	.	.	. (.)	.	.	.	.	.	.	.	.	.	. (Pro)	.	.	.	.
	#03880	.	.	.	.	. (.)	.	.	.	.	.	.	.	.	.	. (Pro)	.	.	.	.
	#03881	-	-	-	-	-	-	-	-	.	.	.	.	.	.	. (Pro)	.	.	.	.
	#03883	.	.	.	.	. (.)	.	.	.	.	.	.	.	.	.	. (Pro)	.	.	.	.
*flatosternata* sp. n.	#04171	.	.	.	.	. (.)	.	.	.	.	.	.	.	.	.	. (Ser)	.	.	.	.
	#04172	.	.	.	.	. (.)	.	.	.	.	.	.	.	.	.	. (Ser)	.	.	.	.
	#04173	.	.	.	.	. (.)	.	.	.	.	.	.	.	.	.	. (Ser)	.	.	.	.
	#04174	-	-	-	-	-	-	-	-	.	.	.	.	.	.	. (Ser)	.	.	.	.
	#04175	.	.	.	.	. (.)	.	.	.	.	.	.	.	.	.	. (Ser)	.	.	.	.
	#04176	.	.	.	.	. (.)	.	.	.	.	.	.	.	.	.	. (Ser)	.	.	.	.
	#04177	-	-	-	-	-	-	-	-	.	.	.	.	.	.	. (Ser)	.	.	.	.
	#04178	.	.	.	.	. (.)	.	.	.	.	.	.	.	.	.	. (Ser)	.	.	.	.
*borneoensis* sp. n.	#03893	.	.	C	C	T (Val)	T	C	C	.	.	.	.	G	.	T (Ile)	.	C	A	G
	#03894	.	.	C	C	T (Val)	T	C	C	.	.	.	.	G	.	T (Ile)	.	C	A	G
	#03895	.	.	C	C	T (Val)	T	C	C	.	.	.	.	G	.	T (Ile)	.	C	A	G
	#03896	.	.	C	C	T (Val)	T	C	C	.	.	.	.	G	.	T (Ile)	.	C	A	G
*javaensis* sp. n.	#03887	C	.	.	.	. (.)	.	.	.	.	C	C	C	.	G	. (.)	C	.	.	.
	#03888	C	.	.	.	. (.)	.	.	.	.	C	C	C	.	G	. (.)	C	.	.	.
	#03889	C	.	.	.	. (.)	.	.	.	.	C	C	C	.	G	. (.)	C	.	.	.
	#03892	C	.	.	-	-	-	-	-	.	C	C	C	.	G	. (.)	C	.	.	.
*sumatraensis* sp. n.	#03890	.	C	.	-	-	-	-	-	C	.	.	.	.	.	. (.)	.	.	.	.
	#03891	.	C	.	.	. (.)	.	.	.	C	.	.	.	.	.	. (.)	.	.	.	.

### Key to the species

In the following key, not only morphological characters but also the selected pure diagnostic nucleotide sites of *COI* and *COII* sequences (Table 5) are used to identify the three cryptic species, *borneoensis* sp. n., *javaensis* sp. n. and *sumatraensis* sp. n.

**Table:** 

1	Postocellar setae absent; ocellar plate granulose; posteromost pseudotrachea of labellum thicker than the others; mid-leg first tarsomere with one subproximal and one apical, short, blackish brown spines, and hindleg first tarsomere with one apical, short spine; prensisetae on surstylus apically blunt (Figs 5–7C); cercus with three prominent setae on anteromedial portion (Figs 5–7B); sclerotized, caudoventral bridge of cerci with a pair of lateral, broad, apically rounded lobes (Figs 5A, 6C, 7C); aedeagus apically hooked (Figs 5G, 6H, 7H); spermathecal capsule pale brown, spherical, slightly broader than long, without apical indentation (Figs 5K, 6L, 7L)	**2**
–	Postocellar setae present (Fig. 4W); ocellar plate smooth, glossy; posteromost pseudotrachea of labellum as thick as the others; first tarsomeres of mid- and hindlegs without short, blackish brown spines; prensisetae on surstylus apically somewhat pointed (Figs 8–10C); cercus with two prominent setae on anteromedial portion (Figs 8–10B); sclerotized, caudoventral bridge of cerci without lateral lobes (Figs 8–10C); aedeagus apically not hooked (Figs 8F, 9F, 10G); spermathecal capsule small, less sclerotized, with apical indentation (Figs 8J, 9J, 10K)	**4**
2	Wing nearly entirely, lightly fuscous, without distinct cloud (Fig. 3C); sclerotized, caudoventral bridge of cerci with small, narrow, apically pointed, median process (Fig. 5A); aedeagus apically curved, hook-like, and finely wrinkled all over, subapically with numerous, coarse serrations, submedially not swollen dorsally (Fig. 5G); spermatheca without distinct introvert (Fig. 5K)	***Di. trilobita* Yang & Gao, sp. n.**
–	Wing largely clouded, except for central pale patch around dm-cu vein and periphery (Fig. 3G,J); sclerotized, caudoventral bridge of cerci without median process (Figs 6C, 7C); aedeagus apically bearing a pair of strongly recurved, smooth hooks, subapically densely spinose, submedially swollen dorsally (Figs 6F,H, 7F,H); spermatheca with introvert 1/5–1/4 as deep as capsule height (Figs 6L, 7L)	**3**
3	Dorsolateral tentorial apodemes nearly parallel in basal half but strongly divergent in distal half (Fig. 4H); tenth sternite mediolaterally with a pair of round depressions (seen in anterior view; Fig. 6E); oviscapt valve 2/5 as broad as long (Fig. 6J)	***Di. heterochroma* Yang & Gao, sp. n.**
–	Dorsolateral tentorial apodemes slightly divergent in basal half but strongly divergent in distal half (Fig. 4M); tenth sternite nearly flat (Fig. 7E); oviscapt valve half as broad as long (Fig. 7J)	***Di. flatosternata* , Yang & Gao, sp. n.**
4	Spermathecal capsule somewhat cylindrical, apically flat; introvert 7/10 as deep as capsule height (Fig. 8J); *COI* = C, C, T, T, C and C at sites 235, 499, 519, 532, 541 and 544, respectively; *COII* = G, T, C, A and G at sites 309, 389, 441, 513 and 636, respectively (Table 5)	***Di. borneoensis* Yang & Gao, sp. n.**
–	Spermathecal capsule somewhat dome-shaped, apically roundish; introvert 2/5 as deep as capsule height (Figs 9J, 10K); *COI* = T, T, C, A, T and T at sites 235, 499, 519, 532, 541 and 544, respectively; *COII* = A, C, T, G and A at sites 309, 389, 441, 513 and 636, respectively (Table 5)	**5**
5	Hypandrium sparsely pubescent in small, medial patch on caudolateral plate (Fig. 9G); *COI* = C and T at sites 142 and 205, respectively; *COII* = T, C, C, C, G and C at sites 18, 69, 270, 303, 381 and 393, respectively (Table 5)	***Di. javaensis* Yang & Gao, sp. n.**
–	Hypandrium without pubescence on caudolateral plate (Fig. 10H); *COI* = T and C at sites 142 and 205, respectively; *COII* = C, T, T, T, A and T at sites 18, 69, 270, 303, 381 and 393, respectively (Table 5)	***Di. sumatraensis* Yang & Gao, sp. n.**

### Description of new species

The characters described above for the genus, the species group, and the key are not referred to in the following descriptions.

#### Dichaetophora trilobita

Taxon classificationAnimaliaORDOFAMILIA

Yang & Gao sp. n.

http://zoobank.org/BAF11C36-EB50-4E98-A8A1-537AE7AD715A

**Figure 4.**
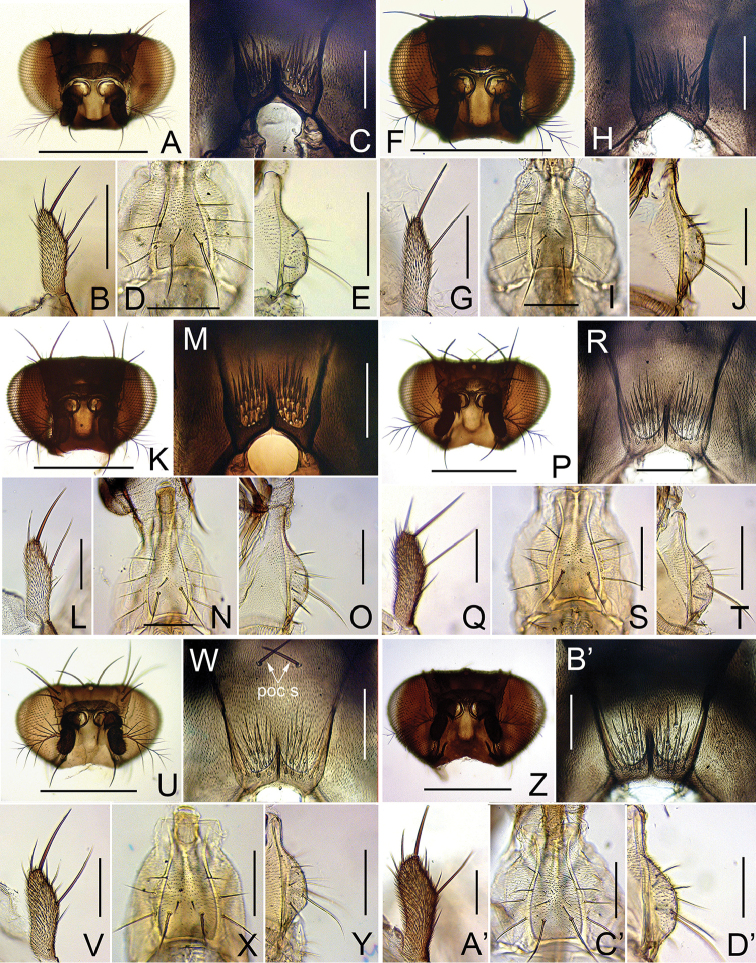
Head (anterior view), postocciput, palpus, and prementum (ventral and lateral view, respectively). **A−E** *Dichaetophora tirlobita* sp. n. (#03877) **F−J** *D. heterochroma* sp. n. (#03879) **K−O** *D. flatosternata* sp. n. (#04172) **P−T** *D. borneoensis* sp. n. (#03895) **U−Y** *D. javaensis* sp. n. (#03892) **Z−D** *D. sumatraensis* sp. n. (#03890). Scale bars: 0.1 mm except for **A, F, K, P, U, Z** (0.5 mm).

**Figure 5.**
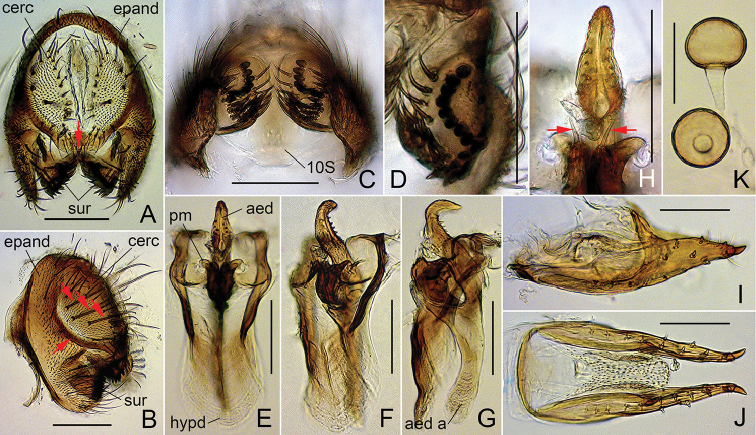
*Dichaetophora trilobita* sp. n. (**A−H** #03877 **I−K** paratype #03878). **A** Periphallic organs (posterior view), with red arrow indicating the median process on the caudoventral bridge of cerci **B** periphallic organs (posterolateral view), with red arrows indicating the prominent setae on the cercus and the anteroventral fusion of cercus (sclerotized, marginal plate) with the epandrium **C** surstyli (ventral view) **D** surstylus (inner side) **E−G** phallic organs (ventral, ventrolateral and lateral view, respectively) **H** paramedian setae (indicated with red arrows), and apical portion of paramere **I, J** oviscapt (lateral and ventral view, respectively) **K** spermathecae. Abbreviations: aed = aedeagus, aed a = aedeagal apodeme, cerc = cercus, epand = epandrium, hypd = hypandrium, pm = paramere, sur = surstylus, 10S = tenth sternite. Scale bars: 0.1 mm.

Figs 3A–D , 4A–E , 5

##### Type material.

Holotype ♂ (#03877): MALAYSIA: Ulu Gombak, Selangor, 8.xii.2013, MJ Toda (UMKL).

Paratypes: same data as holotype (1♀: #03878, UMKL); Kubah, Sarawak, Malaysia, 19.i.1999 (1♂, 1♀, SEHU); Park Headquarters, Mt. Kinabalu, Sabah, Malaysia, 11.iii.2008, MJ Toda (1♂: #03876, KIZ); Poring, Mt. Kinabalu, Sabah, Malaysia, 20.iii.2008, MJ Toda (1♀: #03882, KIZ).

##### Diagnosis.

Postocellar setae absent; wing without distinct cloud (Fig. 3C); sclerotized, caudoventral bridge of cerci with small, narrow, apically pointed, median process and a pair of lateral, broad, apically rounded lobes (Fig. 5A); aedeagus apically curved, hook-like, and finely wrinkled all over, subapically with numerous, coarse serrations, submedially not swollen dorsally (Fig. 5G); spermatheca without distinct introvert (Fig. 5K).

##### Description.

*Head* (Figs 3A, B, 4A–E): First flagellomere grayish yellow. Dorsolateral tentorial apodemes slightly divergent in basal 2/5 but strongly divergent in distal 3/5; supracervical setae 21−23 per side; postocular setae 22–23 per side. Palpus with two subprominent, lateromedial setae. Cibarium with ca. 70 medial and ca. 8 posterior sensilla per side.

*Wings* (Fig. 3C): Veins pale brown to brown. Halter pale gray; stem darker.

*Legs* (Fig. 3A) pale grayish yellow; mid- and hindleg femora distally, foreleg tibia and tarsi proximally to medially darker. Apical setae present on all tibiae; hindleg apical seta short, stout. Mid-leg first tarsomere longer than total length of four succeeding tarsomeres; hindleg first tarsomere as long as total length of four succeeding tarsomeres.

*Male terminalia* (Fig. 5A–H): Epandrium pubescent except for anterior to ventral margin, with 1–3 dorsal and ca. 22 ventral, long setae per side. Surstylus with peg-like prensisetae in sinuate row on caudal margin, 2–5 apically pointed spines and medial patch of pubescence on outer surface and ca. 26 apically pointed spines (ventral ones shorter, thicker and straight, but medial to dorsal ones trichoid and recurved) on inner, caudal portion. Tenth sternite nearly flat. Cercus pubescent except for caudal margin, with 11–12 long setae near dorsal to posterior margin and 7–11 short setae in cluster on ventral portion of sclerotized marginal plate. Paramere with sensilla apically.

*Female terminalia* (Fig. 5I–K): Oviscapt valve dorsomedially narrowly extended, with 3−4 lateral and 9−13 marginal, peg-like ovisensilla.

*Measurements* (in mm): BL (straight distance from anterior edge of pedicel to tip of abdomen) = 2.03 in holotype (1♂ paratype: 2.00; range in 2♀ paratypes: 2.00−2.21), ThL (distance from anterior notal margin to apex of scutellum) = 0.84 (0.82; 0.86−0.88), WL (distance from humeral cross vein to wing apex) = 1.62 (1.69; 1.72−1.73), WW (maximum wing width) = 0.71 (0.72; 0.74−0.79).

*Indices*: FW/HW (frontal width/head width) = 0.53 (range in 1♂, 2♀, or less if noted, paratypes: 0.38−0.50), ch/o (maximum width of gena/maximum diameter of eye) = 0.18 (0.19−0.26), prorb (proclinate orbital seta/posterior reclinate orbital seta in length) = 0.72 (2♀: 0.71−0.80), rcorb (anterior reclinate orbital seta/posterior reclinate orbital seta in length) = 0.30 (2♀: 0.31−0.33), orbito (distance between proclinate and posterior reclinate orbital setae / distance between inner vertical and posterior reclinate orbital setae) = 0.60 (0.58−0.68), dcl (anterior dorsocentral seta/posterior dorsocentral seta in length) = 0.78 (2♀: 0.64−0.74), sctl (basal scutellar seta/apical scutellar seta in length) = 0.67 (1♀: 0.60), sterno (anterior katepisternal seta/posterior katepisternal seta in length) = 0.51 (0.57−0.60), dcp (distance between ipsilateral dorsocentral setae/distance between anterior dorsocentral setae) = 0.49 (0.53−0.60), sctlp (distance between ipsilateral scutellar setae/distance between apical scutellar setae) = 0.79 (0.68−0.77), C (2nd costal section between subcostal break and R_2+3_/3rd costal section between R_2+3_ and R_4+5_) = 1.57 (1.35−1.53), 4c (3rd costal section between R_2+3_ and R_4+5_/M_1_ between r-m and dm-cu) = 1.54 (1.46−1.66), 4v (M_1_ between dm-cu and wing margin/M_1_ between r-m and dm-cu) = 2.19 (2.11−2.33), 5x (CuA_1_ between dm-cu and wing margin/dm-cu between M_1_ and CuA_1_) = 2.19 (2.00−2.38), ac (3rd costal section between R_2+3_ and R_4+5_/distance between distal ends of R_4+5_ and M_1_) = 3.58 (3.66−3.76), M (CuA_1_ between dm-cu and wing margin/M_1_ between r-m and dm-cu) = 0.72 (0.66−0.70), C3F (length of heavy setation in 3rd costal section/length of 3rd costal section) = 0.70 (0.66−0.68).

##### Etymology.

Referring to the trilobed, caudoventral bridge of cercal sclerotized plates.

##### Distribution.

Malaysia (Peninsular Malaysia, Sarawak, Sabah).

#### Dichaetophora heterochroma

Taxon classificationAnimaliaORDOFAMILIA

Yang & Gao sp. n.

http://zoobank.org/4C4A707B-EFFC-474D-A485-381B40B879E8

**Figure 6.**
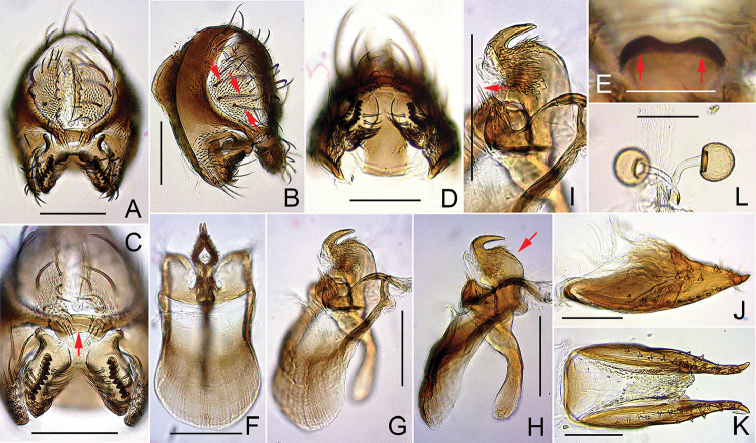
*Dichaetophora heterochroma* sp. n. (**A−I** #03879 **J−L** paratype #03881). **A, B** Periphallic organs (posterior and posterolateral view, respectively) **C** surstyli and cerci, with red arrow indicating the caudoventral bridge of cerci **D, E** tenth sternite (ventral and anterior view, respectively), with red arrows (**E**) indicating a pair of depressions **F−H** phallic organs (ventral, ventrolateral and lateral view, respectively), with red arrow (**H**) indicating the dorsally swollen, submedial portion of aedeagus **I** paramedian setae **J, K** oviscapt (lateral and ventral view, respectively) **L** spermathecae (lateral view). Scale bars: 0.1 mm.

Figs 3E–G , 4F–J , 6

##### Type material.

Holotype ♂ (#03879): MALAYSIA: Poring, Mt. Kinabalu, Sabah, 20.iii.2008, MJ Toda (KPSP).

Paratypes: same data as holotype (1♂, 1♀: #03880, #03881, KIZ; 1♂, SEHU); same data as holotype except for 13.iii.2008 (1♀: #03883, KIZ); same data as holotype except for 19.iii.2008 (1♂, SEHU); same data as holotype except for 3.x.1999 (1♂, SEHU); Mahua, Crocker Range, Sabah, Malaysia, 14.x.1999, MJ Toda (1♀, KPSP; 2♀, SEHU); Ulu Senagang, Crocker Range, Sabah, Malaysia, 18.x.1999, MJ Toda (1♂, 2♀: #03884–3886, ZSM).

##### Diagnosis.

Wing largely clouded, except for central pale patch around dm-cu vein and periphery (Fig. 3G); dorsolateral tentorial apodemes nearly parallel in basal half (Fig. 4H); tenth sternite mediolaterally with a pair of round depressions (seen in anterior view; Fig. 6E); oviscapt valve 2/5 as broad as long (Fig. 6J).

##### Description.

*Head* (Figs 3E, F, 4F–J): First flagellomere grayish yellow. Supracervical setae 15−22 per side; postocular setae 17–21 per side. Palpus with one subprominent lateromedial seta. Cibarium with 78–79 medial and ca. 8 posterior sensilla per side.

*Wings* (Fig. 3G): Veins pale brown to dark brown, but pale within central pale patch. Halter pale gray; stem darker.

*Legs* (Fig. 3E) pale grayish yellow; foreleg coxa, tibia and tarsus, except for fifth tarsomere, dark brown. Mid-leg first tarsomere longer than total length of four succeeding tarsomeres; hindleg first tarsomere as long as total length of four succeeding tarsomeres.

*Male terminalia* (Fig. 6A–I): Epandrium pubescent except for anterior portion, with 3–4 dorsal and 9–12 ventral, long setae per side. Surstylus with prensisetae in nearly straight row on caudal margin, 2–4 apically pointed spines but no pubescence on outer surface and ca. 20 apically pointed, recurved spines on inner, caudal portion. Cercus pubescent except for caudal margin, with 6–12 long setae near dorsal to posterior margin and 6–7 short setae in cluster on ventral portion of sclerotized marginal plate. Paramere with sensilla subapically.

*Female terminalia* (Fig. 6J–L): Oviscapt valve with ca. five lateral and 12–14 marginal, peg-like ovisensilla. Introvert of spermathecal capsule 1/5 as deep as capsule height.

*Measurements* (in mm): BL = 2.18 in holotype (range in 2♂ paratypes: 1.99 –2.21; range in 4♀ paratypes: 2.31–2.77), ThL = 0.79 (0.81–0.85; 0.92 –0.97), WL = 1.64 (1.60–1.64; 1.80 –2.30), WW = 0.68 (0.69–0.70; 0.75 –0.97).

*Indices*: FW/HW = 0.59 (2♂, 4♀, or less if noted, paratypes: 0.50–0.54), ch/o = 0.20 (0.25–0.28), prorb = 0.74 (2♂, 1♀: 0.76–0.87), rcorb = 0.29 (2♂, 3♀: 0.31–0.36), orbito = 0.54 (0.48–0.76), dcl = 0.71 (1♂, 2♀: 0.60–0.73), sctl = n/a (1♂, 3♀: 0.88–0.94), sterno = 0.54 (0.50–0.61), dcp = 0.55 (0.55–0.65), sctlp = 0.62 (0.75–0.95), C = 1.69 (1.99–2.08), 4c = 1.54 (1.41–1.54), 4v = 2.50 (2.28 –2.51), 5x = 1.79 (1.25–1.56), ac = 2.86 (2.38–3.09), M = 0.70 (0.62–0.64), C3F = 0.78 (0.73–0.87).

##### Etymology.

Referring to the heterochromatic legs.

##### Distribution.

Malaysia (Sabah).

#### Dichaetophora flatosternata

Taxon classificationAnimaliaORDOFAMILIA

Yang & Gao sp. n.

http://zoobank.org/2BAD2A68-B183-4781-AF74-A103DEF614F4

**Figure 7.**
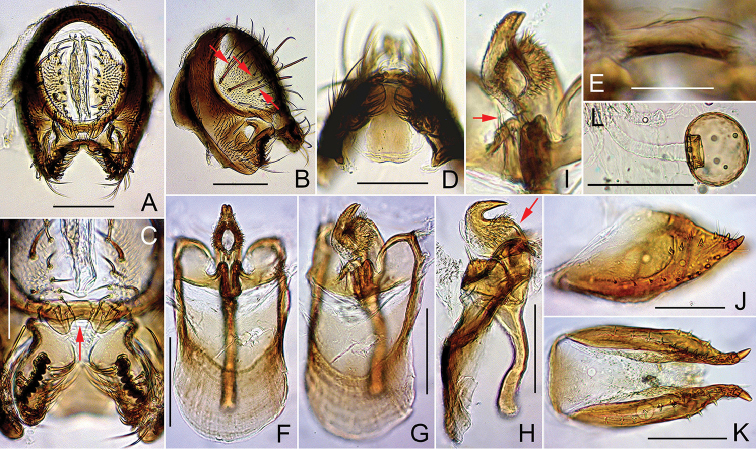
*Dichaetophora flatosternata* sp. n. (**A−I** #04172 **J−L** paratype #04177). **A, B** Periphallic organs (posterior and posterolateral view, respectively) **C** surstyli and cerci **D, E** tenth sternite (ventral and anterior view, respectively) **F−H** phallic organs (ventral, ventrolateral and lateral view, respectively) **I** paramedian setae **J, K** oviscapt (lateral and ventral view, respectively) **L** spermatheca (lateral view). Scale bars: 0.1 mm.

Figs 3H–J , 4K–O , 7

##### Type material.

Holotype. ♂ (#04172), CHINA: Mengyuan Substation, Mengla Station, Xishuangbanna National Nature Reserve, Guanlei, Mengla, Xishuangbanna, Yunnan, 14–15.xi.2012, JJ Gao (KIZ).

Paratypes: same data as holotype (5♂, 2♀: #04171, #04173–4178, KIZ, SEHU).

##### Diagnosis.

Wing largely clouded, except for central pale patch around dm-cu vein and periphery (Fig. 3J); dorsolateral tentorial apodemes slightly divergent in basal half (Fig. 4M); tenth sternite nearly flat (Fig. 7E); oviscapt valve half as broad as long (Fig. 7J).

##### Description.

*Head* (Figs 3H, I, 4K–O): First flagellomere grayish yellow. Supercervical setae 17−18 per side; postocular setae 20–22 per side. Palpus with one subprominent lateromedial seta. Cibarium with 77–79 medial and ca. 10 posterior sensilla per side.

*Wings* (Fig. 3J): Veins pale brown to dark brown, but pale within central pale patch. Halter pale gray; stem darker.

*Legs* (Fig. 3H) pale grayish yellow; foreleg tibia and tarsus, mid-leg femur and distal portion of hindleg femur darker. Hindleg tibia lacking apical seta. Mid-leg first tarsomere longer than total length of four succeeding tarsomeres; hindleg first tarsomere as long as total length of four succeeding tarsomeres.

*Male terminalia* (Fig. 7A–I): Epandrium pubescent except for anterior portion, with ca. three dorsal and 10–11 ventral, long setae per side. Surstylus with prensisetae in slightly concave row on caudal margin, 2–3 apically pointed spines but no pubescence on outer surface and ca. 16 apically pointed, recurved spines on inner, caudal portion. Cercus pubescent except for caudal margin, with 10–13 long setae near dorsal to posterior margin and 8–10 short setae in cluster on ventral portion of sclerotized marginal plate. Paramere with sensilla subapically.

*Female terminalia* (Fig. 7J–L): Oviscapt valve with ca. four lateral and 12–13 marginal, peg-like ovisensilla. Introvert of spermathecal capsule 1/4 as deep as capsule height.

*Measurements* (in mm): BL = 2.10 in holotype (range in 5♂ paratype: 2.08–2.32; range in 2♀ paratypes: 2.21 –2.31), ThL = 0.87 (0.83–0.88; 0.83 –0.93), WL = 1.78 (1.67–1.81; 1.71 –1.81), WW = 0.81 (1.79–1.82; 0.81–0.83).

*Indices*: FW/HW = 0.54 (5♂, 2♀, or less if noted, paratypes: 0.49–0.54), ch/o = 0.23 (0.27–0.29), prorb = 0.71 (0.79–0.87), rcorb = n/a (0.33–0.39), orbito = 0.54 (0.55–0.74), dcl = 0.64 (0.62–0.78), sctl = n/a (4♂, 2♀: 0.89–0.97), sterno = 0.65 (0.54–0.63), dcp = 0.55 (0.54–0.62), sctlp = 0.73 (0.74–0.80), C = 1.53 (1.63–1.71), 4c = 1.45 (1.22–1.36), 4v = 1.96 (1.42–1.60), 5x = 1.89 (1.79–1.94), ac = 3.04 (2.53–2.76), M = 0.64 (0.57–0.64), C3F = 0.69 (0.62–0.71).

##### Etymology.

Referring to the flat male tenth sternite.

##### Distribution.

China (Yunnan).

#### Dichaetophora borneoensis

Taxon classificationAnimaliaORDOFAMILIA

Yang & Gao sp. n.

http://zoobank.org/20778512-6C3C-4BC2-99A6-83A02C6FBCC4

**Figure 8.**
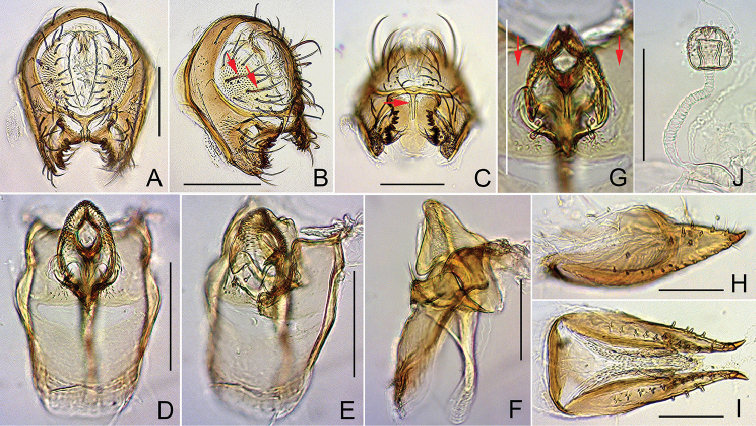
*Dichaetophora borneoensis* sp. n. (**A−G** #03895 **H−J** paratype #03894). **A, B** Periphallic organs (posterior and posterolateral view, respectively) **C** surstyli and cerci, with red arrow indicating the median, elongated process on the caudoventral bridge of cerci **D−F** phallic organs (ventral, ventrolateral and lateral view, respectively) **G** distal portion of hypandrium (posteroventral view), showing the caudolateral plates not pubescent (red arrows) and the paramedian setae **H, I** oviscapt (lateral and ventral view, respectively) **J** spermatheca (lateral view). Scale bars: 0.1 mm.

Figs 3K–M , 4P–T , 8

##### Type material.

Holotype ♂ (#03895), MALAYSIA: Park Headquarters, Mt. Kinabalu, Sabah, 16.viii.2011, K Akutsu (KPSP).

Paratypes: same data as holotype except for 17.viii.2011 (1♂: #03896, KIZ); same data as holotype except for 11.iii.2008 (2♀: #03893, #03894, KIZ); same data as holotype except for 2.i.1999, MJ Toda (8♂, 2♀, SEHU; 1♀, KPSP; 1♀, ZSM); Poring, Mt. Kinabalu, Sabah, Malaysia, 28.xii.1998 (1♂); Mahua, Crocker Range, Sabah, Malaysia, 14.x.1999, MJ Toda (1♂, ZSM).

##### Diagnosis.

Postocellar setae present; spermathecal capsule somewhat cylindrical, apically flat; introvert 7/10 as deep as capsule height (Fig. 8J); *COI* = C, C, T, T, C and C at sites 235, 499, 519, 532, 541 and 544, respectively; *COII* = G, T, C, A and G at sites 309, 389, 441, 513 and 636, respectively (Table 5).

##### Description.

*Head* (Figs 3K, L, 4P–T): First flagellomere grayish brown. Dorsolateral tentorial apodemes slightly divergent in basal 2/5 but strongly divergent in distal 3/5; supracervical setae 21−25 per side; postocular setae 23−24 per side. Palpus with one subprominent, lateromedial seta. Cibarium with ca. 80 medial and 9−12 posterior sensilla per side.

*Wings* (Fig. 3M) slightly fuscous; veins yellowish brown to brown. Halter and stem gray.

*Legs* (Fig. 3K): All femora blackish brown; foreleg coxa, tibia and tarsus (except 5^th^ tarsomere) grayish brown; rest grayish yellow. Apical setae present on foreleg and mid-leg tibiae. Mid-leg first tarsomere as long as total length of four succeeding tarsomeres; hindleg first tarsomere longer than total length of four succeeding tarsomeres.

*Male terminalia* (Fig. 8A–G): Epandrium pubescent except for anterior margin, with 1–2 dorsal and 7–11 ventral, long setae per side. Surstylus with 1–3 short spines and medial patch of pubescence on outer surface and 7–10 recurved spines on inner, caudal portion. Tenth sternite lingulate, slightly curved. Cercus pubescent except for dorsal to caudal margin, with 15–17 long setae distributed nearly all over; sclerotized, caudoventral bridge of cerci medially with narrowly elongated process. Paramere somewhat triangular in lateral view, apically round, with sensilla on inner surface. Aedeagus apically trilobed, densely hirsute on outer lobes, dorsosubapically with a pair of small, marginally serrated flaps.

*Female terminalia* (Fig. 8H–J): Oviscapt valve dorsomedially narrowly extended, with 4−5 lateral and 12−15 marginal, peg-like ovisensilla. Spermathecal capsule with fine spinules on distal half of outer surface.

*Measurements* (in mm): BL = 2.42 in holotype (1♂ paratype: 2.10; range in 2♀ paratypes: 2.34–2.57), ThL = 1.01 (0.87; 0.93–0.96), WL = 2.19 (1.84; 1.82–2.14), WW = 0.97 (0.85; 0.92–0.94).

*Indices*: FW/HW = 0.50 (1♂, 2♀, or less if noted, paratypes: 0.48–0.51), ch/o = 0.19 (0.25–0.29), prorb = 0.66 (1♂, 1♀: 0.63–0.73), rcorb = 0.23 (1♂, 1♀: 0.24–0.30), orbito = 0.62 (0.53–0.60), dcl = 0.76 (1♀: 0.77), sctl = 0.86 (0.84–0.87), sterno = 0.59 (1♂, 1♀: 0.58–0.62), dcp = 0.49 (0.49–0.52), sctlp = 0.90 (0.84–0.89), C = 1.82 (1.82–2.01), 4c = 1.47 (1.31–1.51), 4v = 2.51 (2.22–2.70), 5x = 2.16 (2.11–2.32), ac = 2.81 (2.88–3.15), M = 0.79 (0.71–0.87), C3F = 0.59 (0.57–0.63).

##### Etymology.

Pertaining to the type locality.

##### Distribution.

Malaysia (Sabah).

#### Dichaetophora javaensis

Taxon classificationAnimaliaORDOFAMILIA

Yang & Gao sp. n.

http://zoobank.org/09DA1C1A-C0A2-4025-99FB-6AAAC123F295

**Figure 9.**
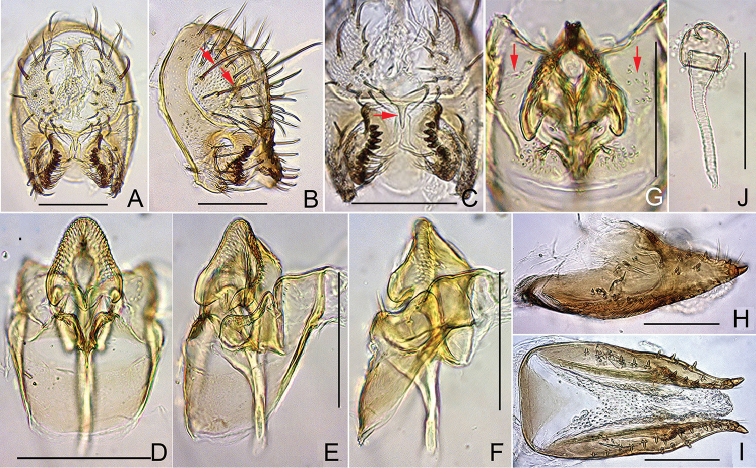
*Dichaetophora javaensis* sp. n. (**A−G** #03892 **H−J** paratype #03888). **A, B** Periphallic organs (posterior and posterolateral view, respectively) **C** surstyli and ventral portions of cerci **D−F** phallic organs (ventral, ventrolateral and lateral view, respectively) **G** distal portion of hypandrium (posteroventral view), showing a pair of small patches of sparse pubescence on the caudolateral plates (red arrows) and the paramedian setae **H, I** oviscapt (lateral and ventral view, respectively) **J** spermatheca (lateral view). Scale bars: 0.1 mm.

Figs 3N–P , 4U–Y , 9

##### Type material.

Holotype. ♂ (#03892), INDONESIA: Cikaniki, Mt. Halimun, West Java, 7.x.2009, MJ Toda (MZB).

Paratypes: same as holotype except for 6.xi.2009 (1♀: #03887, MZB; 2♀: #03888–9, KIZ); same as holotype except for 10.xi.2009 (1♀, SEHU); Cibodas, West Java, Indonesia, 16.xi.2013, MJ Toda (1♂, SEHU); Mt. Patuha, Sugihmukti, West Java, Indonesia, 13.x.2004 (1♂, SEHU).

##### Diagnosis.

Postocellar setae present; spermathecal capsule somewhat dome-shaped, apically roundish; introvert 2/5 as deep as capsule height (Fig. 9J); hypandrium sparsely pubescent in small, medial patch on caudolateral plate (Fig. 9G); *COI* = C and T at sites 142 and 205, respectively; *COII* = T, C, C, C, G and C at sites 18, 69, 270, 303, 381 and 393, respectively (Table 5).

##### Description.

*Head* (Figs 3N, O, 4U–Y): First flagellomere grayish brown. Dorsolateral tentorial apodemes slightly divergent in basal 2/5 but strongly divergent in distal 3/5; supercervical setae 18−22 per side; postocular setae 19 per side. Palpus with one subprominent, lateromedial seta. Cibarium with ca. 90 medial and 10−12 posterior sensilla per side.

*Wings* (Fig. 3P) slightly fuscous; veins yellowish brown to brown. Halter and stem gray.

*Legs* (Fig. 3N): All femora blackish brown; foreleg coxa, tibia and tarsus (except 5^th^ tarsomere) grayish brown; rest grayish yellow. Apical setae present on foreleg and mid-leg tibiae. Mid-leg first tarsomere as long as total length of four succeeding tarsomeres; hindleg first tarsomere longer than total length of four succeeding tarsomeres.

*Male terminalia* (Fig. 9A–G): Epandrium pubescent except for anterior margin, with 2–3 dorsal and 9–10 ventral, long setae per side. Surstylus with 2–3 short spines and medial patch of pubescence on outer surface and 10–11 recurved spines on inner, caudal portion. Tenth sternite lingulate, slightly curved. Cercus pubescent except for dorsal to caudal margin, with 14–17 long setae; sclerotized, caudoventral bridge of cerci medially with narrowly elongated process. Paramere somewhat triangular in lateral view, apically round, with sensilla on inner surface. Aedeagus apically trilobed, densely hirsute on outer lobes, dorsosubapically with a pair of small, marginally serrated flaps.

*Female terminalia* (Fig. 9H–J): Oviscapt valve dorsomedially narrowly extended, with 4−5 lateral and 12−15 marginal, peg-like ovisensilla. Spermathecal capsule without spinules on outer surface.

*Measurements* (in mm): BL = 1.94 in holotype (range in 3♀ paratypes: 2.08–2.50), ThL = 0.86 (0.97–1.03), WL = 1.81 (1.99–2.07), WW = 0.82 (0.86–0.92).

*Indices*: FW/HW = 0.56 (3♀, or less if noted, paratypes: 0.49–0.52), ch/o = 0.21 (0.34–0.41), prorb = 0.76 (0.72–0.79), rcorb = 0.24 (0.29–0.31), orbito = 0.60 (0.54–0.69), dcl = 0.74 (1♀: 0.71), sctl = 0.81(1♀: 0.81), sterno = 0.60 (0.50–0.54), dcp = 0.52 (0.53–0.60), sctlp = 0.71 (0.52–0.71), C = 1.85 (1.78–1.98), 4c = 1.54 (1.35–1.40), 4v = 2.62 (2.26–2.28), 5x = 2.28 (1.73–1.91), ac = 3.03 (2.95–3.24), M = 0.76 (0.71–0.90), C3F = 0.58 (0.50–0.60).

##### Etymology.

Pertaining to the type locality.

##### Distribution.

Indonesia (West Java).

#### Dichaetophora sumatraensis

Taxon classificationAnimaliaORDOFAMILIA

Yang & Gao sp. n.

http://zoobank.org/8F7EB4F2-3B81-4D2F-9AAC-3ABD144F8BC7

Figs 3Q–S , 4Z–D ’, 10

##### Type material.

Holotype ♂ (#03890): INDONESIA: Mt. Kerinci., Jambi, Sumatra, 7.x.2004, MJ Toda (MZB).

Paratypes: same as holotype (1♀: #03891, MZB); same as holotype except for 6.x.2004 (1♀, SEHU).

##### Diagnosis.

**Figure 10.**
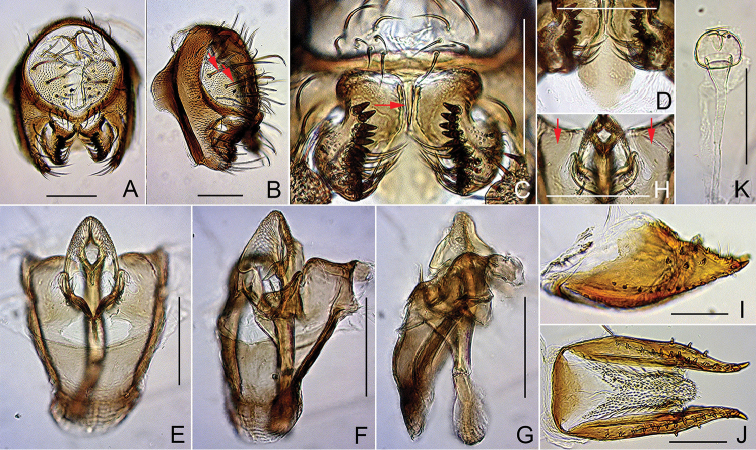
*Dichaetophora sumatraensis* sp. n. (**A−H** #03890 **I−K** paratype #03891). **A, B** Periphallic organs (posterior and posterolateral view, respectively) **C** surstyli and ventral portions of cerci **D** tenth sternite (posteroventral view) **E−G** phallic organs (ventral, ventrolateral and lateral view, respectively) **H** distal portion of hypandrium (posteroventral view), showing the caudolateral plates not pubescent (red arrows) and the paramedian setae **I, J** oviscapt (lateral and ventral view, respectively) **K** spermatheca (lateral view). Scale bars: 0.1 mm.

Postocellar setae present; spermathecal capsule somewhat dome-shaped, apically roundish; introvert 2/5 as deep as capsule height (Fig. 10K); hypandrium without pubescence on caudolateral plate (Fig. 10H); *COI* = T and C at sites 142 and 205, respectively; *COII* = C, T, T, T, A and T at sites 18, 69, 270, 303, 381 and 393, respectively (Table 5).

##### Description.

*Head* (Figs 3Q, R, 4Z–D’): First flagellomere grayish brown. Dorsolateral tentorial apodemes slightly divergent in basal 2/5 but strongly divergent in distal 3/5; supercervical setae 19−22 per side; postocular setae 20−24 per side. Palpus with one subprominent, lateromedial seta. Cibarium with numerous (> 60) medial and 8−9 posterior sensilla per side.

*Wing* (Fig. 3S) slightly fuscous; veins yellowish brown to brown. Halter and stem gray.

*Legs* (Fig. 3Q): All femora blackish brown; foreleg coxa, tibia and tarsus (except 5^th^ tarsomere) grayish brown; rest grayish yellow. Apical setae present on foreleg and mid-leg tibiae. Mid-leg first tarsomere as long as total length of four succeeding tarsomeres; hindleg first tarsomere longer than total length of four succeeding tarsomeres.

*Male terminalia* (Fig. 10A–H): Epandrium pubescent except for anterior margin, with three dorsal and 11–13 ventral, long setae per side. Surstylus with 9–10 prensisetae caudal margin, 2–3 short spines and medial patch of pubescence on outer surface and ca. 15 recurved spines on inner, caudal portion. Tenth sternite lingulate, slightly curved. Cercus pubescent except for dorsal to caudal margin, with 20–21 long setae; sclerotized, caudoventral bridge of cerci medially with narrowly elongated process. Paramere somewhat triangular in lateral view, apically round, with sensilla on inner surface. Aedeagus apically trilobed, densely hirsute on outer lobes, dorsosubapically with a pair of small, marginally serrated flaps.

*Female terminalia* (Fig. 10I–K): Oviscapt valve dorsomedially narrowly extended, with 5−6 lateral and 14−17 marginal, peg-like ovisensilla. Spermathecal capsule without spinules on outer surface.

*Measurements* (in mm): BL = 2.33 in holotype (1♀ paratype: 2.70), ThL = 1.04 (1.13), WL = 2.17 (2.46), WW = 1.10 (1.00).

*Indices*: FW/HW = 0.36 (1♀ paratype: 0.40), ch/o = 0.39 (0.33), prorb = n/a (n/a), rcorb = n/a(n/a), orbito = 0.75 (0.78), dcl = 0.72 (n/a), sctl = n/a (n/a), sterno = n/a (0.61), dcp = 0.62 (0.60), sctlp = 0.80 (0.75), C = 1.90 (2.00), 4c = 1.29 (1.34), 4v = 2.18 (2.23), 5x = 2.18 (1.84), ac = 2.37 (2.57), M = 0.79 (0.75), C3F = 0.58 (0.60).

##### Etymology.

Pertaining to the type locality.

##### Distribution.

Indonesia (Sumatra).

##### Remarks.

The last three species somewhat resemble *trilobita* sp. n. in having the following morphological characters: wing without distinct, dark cloud; surstylus with medial patch of pubescence on outer surface; sclerotized, caudoventral bridge of cerci with narrow, median process; and oviscapt valve dorsomedially narrowly extended. However, the three species are very hard to distinguish from each other because of their least morphological differentiation. To overcome this difficulty, we employed 19 nucleotide sites of *COI* and *COII* genes as molecular diagnostic characters to identify these cryptic species (Table 5). Nucleotide substitutions at two of these sites are nonsynonymous, i.e., causing changes of amino acids, and specific to *borneoensis* sp. n. (Table 5), thus providing more reliable (less changeable) characters for this species.

## Supplementary Material

XML Treatment for Dichaetophora trilobita

XML Treatment for Dichaetophora heterochroma

XML Treatment for Dichaetophora flatosternata

XML Treatment for Dichaetophora borneoensis

XML Treatment for Dichaetophora javaensis

XML Treatment for Dichaetophora sumatraensis

## References

[B1] Burla H (1954) Zur Kenntnis der Drosophiliden der Elfenbeinkiiste (Franzosisch West-Afrika). Revue Suisse de Zoologie 61: 1–218. https://doi.org/10.5962/bhl.part.75413

[B2] Duda O (1940) Revision der afrikanischen Drosophiliden (Diptera). II. Annales Historico-Naturales Musei Nationalis Hungarici 33: 19–53.

[B3] DeSalle R Egan MG Siddall M (2005)

[B4] Gao JJ Watabe H Aotsuka T Pang JF Zhang YP (2007) Molecular phylogeny of the *Drosophila obscura* species group, with emphasis on the Old World species. BMC Evolutionary Biology 7: article no. 87. https://doi.org/10.1186/1471-2148-7-87 10.1186/1471-2148-7-87 PMC1904182 17555574

[B5] Grimaldi DA (1990) A phylogenetic, revised classification of genera in the Drosophilidae (Diptera). Bulletin of the American Museum of Natural History 197: 1–139.

[B6] Folmer O Black M Hoeh W Lutz R Vrijenhoek R (1994) DNA primers for amplification of mitochondrial cytochrome *c* oxidase subunit I for diverse metazoan invertebrates. Molecular Marine Biology and Biotechnology 3: 294–299. 7881515

[B7] Hu YG Toda MJ (2002) Cladistic analysis of the genus *Dichaetophora* Duda (Diptera: Drosophilidae) and a revised classification. Insect Systematics and Evolution 33: 91–102. https://doi.org/10.1163/187631202X00064

[B8] Hu YG Toda MJ (2005) A new species group in the genus *Dichaetophora* Duda (Diptera: Drosophilidae) based on a phylogenetic analysis, with descriptions of four new species from China. Zoological Science 22: 1266–1276. http://dx.doi.org/10.2108/zsj.22.1265 10.2108/zsj.22.1265 16357475

[B9] Kumar S Stecher G Tamura K (2016) MEGA7: Molecular evolutionary genetics analysis version 7.0 for bigger datasets. Molecular Biology and Evolution 33: 1870–1874. https://doi.org/10.1093/molbev/msw054 27004904 10.1093/molbev/msw054 PMC8210823

[B10] Li NN Toda MJ Fu Z Li SH Gao JJ (2014) Taxonomy of the *Colocasiomyia gigantea* species group (Diptera, Drosophilidae), with descriptions of four new species from Yunnan, China. ZooKeys 406: 41–64. https://doi.org/10.3897/zookeys.406.7176 10.3897/zookeys.406.7176 PMC4023246 24843281

[B11] McAlpine JF (1981) Morphology and terminology: adults. In: McAlpine JF Peterson BV Shewell GE Teskey HJ Vockeroth JR Wood DM (Eds) Manual of Nearctic Diptera, Vol 1. Biosystematics Research Institute, Ottawa, 9–63.

[B12] Meier R Zhang G Ali F (2008) The use of mean instead of smallest interspecific distances exaggerates the size of the “barcoding gap” and leads to misidentification. Systematic Biology 57: 809–813. https://doi.org/10.1080/10635150802406343 18853366 10.1080/10635150802406343

[B13] Ronquist F Huelsenbeck JP (2003) MrBayes 3: Bayesian phylogenetic inference under mixed models. Bioinformatics 19: 1572–1574. https://doi.org/10.1093/bioinformatics/btg180 12912839 10.1093/bioinformatics/btg180

[B14] Sarkar IN Thornton JW Planet PJ Figurski DH Schierwater B DeSalle R (2002) An automated phylogenetic key for classifying homeoboxes. Molecular Phylogenetics and Evolution 24: 388–399. https://doi.org/10.1016/S1055-7903(02)00259-2 12220982 10.1016/s1055-7903(02)00259-2

[B15] Simon C Frati F Beckenbach A Crespi B Liu H Flook P (1994) Evolution, weighting, and phylogenetic utility of mitochondrial gene sequences and a compilation of conserved polymerase chain reaction primers. Annals of the Entomological Society of America 87: 651–701. https://doi.org/10.1093/aesa/87.6.651

[B16] Srivathsan A Meier R (2012) On the inappropriate use of Kimura-2-parameter (K2P) divergences in the DNA-barcoding literature. Cladistics 28: 190–194. https://doi.org/10.1111/j.1096-0031.2011.00370.x 10.1111/j.1096-0031.2011.00370.x 34861755

[B17] Zhang WX Toda MJ (1992) A new species-subgroup of the *Drosophila immigrans* species-group, with description of two new species from China and revision of taxonomic terminology. Japanese Journal of Entomology 60: 839–850.

